# Estimation of Physiological Tremor from Accelerometers for Real-Time Applications

**DOI:** 10.3390/s110303020

**Published:** 2011-03-07

**Authors:** Kalyana C. Veluvolu, Wei Tech Ang

**Affiliations:** 1 School of Electronics Engineering, Kyungpook National University, Daegu, Korea; 2 School of Mechanical and Aerospace Engineering, Nanyang Technological University, Singapore; E-Mail: wtang@ntu.edu.sg

**Keywords:** tremor, inertial sensors, BMFLC, Kalman filter, real-time estimation

## Abstract

Accurate filtering of physiological tremor is extremely important in robotics assisted surgical instruments and procedures. This paper focuses on developing single stage robust algorithms for accurate tremor filtering with accelerometers for real-time applications. Existing methods rely on estimating the tremor under the assumption that it has a single dominant frequency. Our time-frequency analysis on physiological tremor data revealed that tremor contains multiple dominant frequencies over the entire duration rather than a single dominant frequency. In this paper, the existing methods for tremor filtering are reviewed and two improved algorithms are presented. A comparative study is conducted on all the estimation methods with tremor data from microsurgeons and novice subjects under different conditions. Our results showed that the new improved algorithms performed better than the existing algorithms for tremor estimation. A procedure to separate the intended motion/drift from the tremor component is formulated.

## 1. Introduction

Tremor is defined as “a rhythmic, involuntary movement of a body part” [1,2]. Tremor exists in all humans in small magnitude and is considered as physiological tremor. However, there exists pathologies with very disabling forms of tremor caused by movement disorders called as pathological tremors. They are classified by position/motor behavior. Accordingly, pathological tremor can be classified into three categories: rest, postural and kinetic tremor [2]. Physiological tremor has different aetiology compared to pathological tremor and manifests differently in terms of amplitude and frequency [3–5].

The general assumption is that tremor has a single dominant frequency [3,5]. Pathological tremor (patients with essential tremor and Parkinson disease) tend to display tremor with a dominant frequency [5–8]. The frequency of the pathological tremor tends to remain constant with slight variations [5]. The amplitude of the physiological tremor is much lower than the pathological tremor and has different frequency bands. Recent results [9] for subjects with physiological tremor contradict the general assumption and suggest that tremor parameters (like amplitude, frequency, bandwidth) largely vary from subject to subject. In [9], it was shown that for physiological tremor has multiple dominant frequencies with 3–4 Hz bandwidth.

Physiological hand tremor lies in the band of 8–12 Hz with an amplitude of 50 *μm* and can be approximated by a sinusoidal movement [1,3]. Physiological tremor leads to an intolerable imprecision of the surgical procedure (e.g., vitreoretinal surgery) which require a positioning accuracy of about 10 *μm* [10]. To compensate physiological tremor, robotics assisted surgical procedures have received significant attention [11–13]. In [11,12] a robotic handheld instrument to cancel physiological tremor of surgeon in vitreoretinal microsurgery was implemented. The robotic instrument has to estimate the tremor motion and generate an out-of-phase movement to cancel it in the real-time. The tip of the micron will be unaffected by the tremor motion of the surgeon. MEMS accelerometers constitutes the most common for tremor sensing in robotics assisted procedures [14,15].

Physiological tremor presents a technical challenge because of the high frequency band and its application in real-time. The error compensation control loop has to be executed in real-time. The system has to sense the tremor motion, distinguish between voluntary and undesired components, and generate an out-of-phase movement of the effector (hardware or software) to nullify the erroneous part, all in one sampling cycle. This approach will only work when there is a distinctive and accurate separation between the desired and unwanted motion. For example, dominant frequency of physiological hand tremor lies in the band of 6–15 Hz while hand movement of surgeon during microsurgery is almost always less than 0.5–1 Hz. Due to presence of accelerometers in tremor sensing equipment, physiological tremor filtering is more challenging with the presence of drift, noise and gravity in acceleration measurements [14].

Although linear filters [16,17] are successful in compensating tremor, the inherent time delay [18] is a major drawback where zero-phase filtering is required. In [19], it was shown that delay as small as 30 ms may degrade performance in human-machine control applications. Effective tremor compensation requires zero-phase lag in the filtering process so that the filtered tremor signal can be used to generate an opposing motion to tremor in real-time. To overcome the problems with delays, adaptive algorithms like Weighted-frequency Fourier linear combiner (WFLC) and Band limited multiple Fourier linear combiner (BMFLC) are developed. Weighted-frequency Fourier linear combiner (WFLC) [20] is an adaptive algorithm which models any quasi-periodic signal as a modulating sinusoid, and tracks its frequency, amplitude and phase. WFLC incorporates frequency adaptation procedure into Fourier Linear Combiner (FLC) [21]. Main drawback of WFLC lies in tracking signals with multiple dominant frequencies, for example physiological tremor display multiple dominant frequencies [9]. Any presence of low-frequency component or high frequency noise affects the frequency adaptation, and compels the use of pre-filtering (band-pass filter) that introduces delay into the filtering process. In [4], pre-filtering state was employed to eliminate voluntary motion and tremor was modeled with WFLC for data sensed from gyroscopes for joint rotation. Recently, a two stage algorithm was developed for tremor estimation with WFLC and Kalman Filter for pathological tremor [7] with gyroscopes. Most of the techniques discussed are not ideal for robotics related tremor cancellation due to involvement of accelerometers that only provide acceleration of the tremor motion.

Band limited multiple Fourier linear combiner (BMFLC) [9,22] is also an adaptive algorithm developed to track multiple dominant frequencies in tremor for accurate tremor filtering. The adaption process is achieved using least mean square (LMS) optimization similar to WFLC and FLC. As physiological tremor has multiple dominant frequencies [9], BMFLC-based algorithms should perform better than WFLC-based algorithms. As the frequency components in BMFLC are constant, analytical integration can be employed to obtain the displacement from acceleration. In a single stage, BMFLC can also separate voluntary and involuntary motion and can provide the tremor signal in the displacement domain. Due to this reason, it becomes an ideal choice for tremor filtering when data is sensed with accelerometers.

In this paper, a study is conducted on 6 micro surgeons and 6 healthy subjects to analyze time-frequency tremor characteristics. Existing methods on tremor estimation are first reviewed and the two improved methods are discussed. We improve the existing BMFLC algorithm by modifying the adaption procedure. Instead of relying on LMS for adaptation, we combine BMFLC with recursive least squares (RLS) and Kalman Filter to develop two new methods for accurate tremor estimation. All the existing and proposed methods are reviewed for performance on the data collected. The estimation accuracy is validated over several trails of data to show the effectiveness of the proposed methods.

## 2. Tremor Data and Characteristics

**Figure 1.**
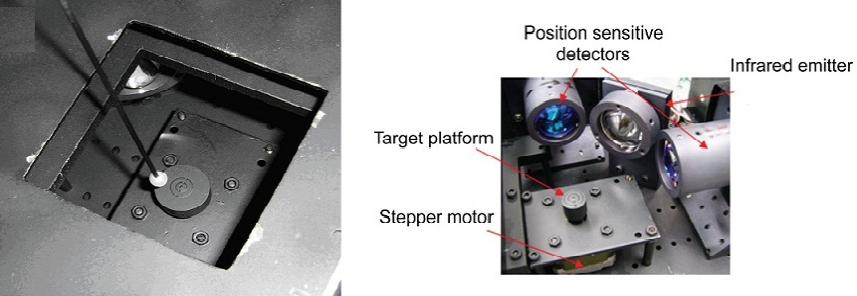
Micro Motion Sensing System (M^2^S^2^) setup.

Tremor recordings are performed through the Micro Motion Sensing System (M^2^S^2^) [23,24]. The M^2^S^2^ consists of a pair of orthogonally placed position sensitive detectors (PSD) and an infra-red (IR) diode to track the 3D displacement of the tip of microsurgical instrument in real-time. The IR diode is used to illuminate the workspace. A ball is attached to the tip of an intraocular shaft to reflect IR rays onto the PSDs. Experimental setup is shown in Figure 1. Instrument tip position is then calculated from the centroid position of the light falling on the PSDs. The resolution, minimum accuracy and sampling rate of the M^2^S^2^ are 0.7 *μm*, 98%, and 250 Hz respectively [23].

The tremor data recorded from 6 healthy subjects and 6 microsurgeons is considered for analysis in this paper. All subjects gave informed consent prior to the test and reported no physical or cognitive impairments. The subjects had their wrists rested on a small platform of the (M^2^S^2^) and were asked to take a comfortable seating position. They had to hold the stylus between their index finger and thumb in order to ensure that all subjects have similar grip across trials. The tip of the stylus was pointed near the center of the M^2^S^2^ workspace. Two types of tasks are performed by the subjects:

1. *Stationary Task:* In this task, subjects are instructed to point the laser light at the center point of the platform with the stylus provided for 30 s duration.
2. *Tracing Task:* In this task, subjects trace the circumference of a circular path on the platform for 30s, with the speed that is realistic for surgical micro manipulation tasks.

The subjects performed two trials for each task with approximately one minute break between each trail.

### 2.1. Time-Frequency Characteristics of Tremor

In [9], it was shown that the physiological tremor characteristics varied between groups of surgeons and novice subjects. Results showed that existence of several dominant tremor frequencies in the tremor band of healthy subjects. The awareness of the tests and procedures likely affect the tremor band as surgeons were able to control the tremor amplitude. It was further quantitatively shown in [9], that the tremor amplitude of surgeons is lower than for the novices.

The analysis of tremor frequency characteristics can be performed with the single sided amplitude spectrum. Using the amplitude spectra, the dominant frequencies and the bandwidth of the tremor can be identified. However, the time-frequency characteristics of the tremor cannot be quantified with the amplitude spectrum. Existence of multiple peaks in amplitude spectrum of healthy subjects can also be related to dynamic changes in tremor frequency in the given band. It was not clear whether the single dominant frequency changes or there exists multiple dominant frequencies at any given time instant. To further analyze the tremor characteristics, we employ the BMFLC [9,25] to obtain the time-frequency map. By construction, BMFLC divides the time domain signal into individual time-frequency components there by providing a high-resolution time-frequency map of the signal in a given band of interest.

**Figure 2.**
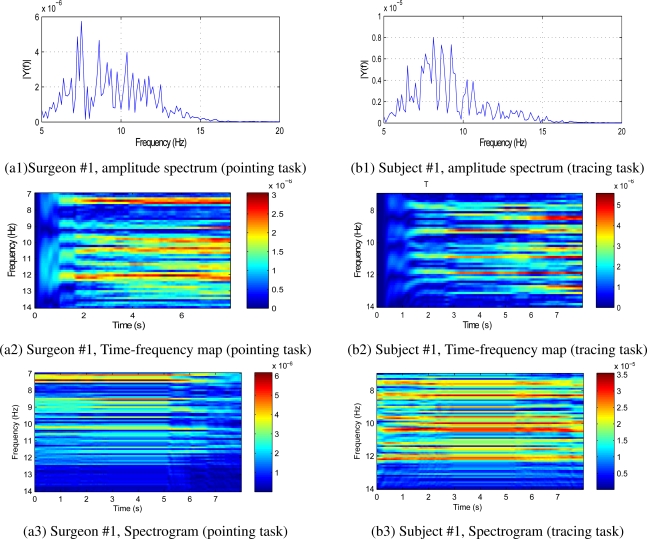
Time-Frequency mapping of surgeon #1 and novice subject #1 in the band of 7–14 Hz.

To study the time-frequency characteristics of tremor, the data of 6 microsurgeons and 6 healthy subjects are analyzed with BMFLC. For illustration, time-frequency mapping, FFT spectrum analyzer and spectrogram for surgeon #1 and novice subject #1 are shown in the Figure 2. Observation of tremor amplitude spectrum and BMFLC time-frequency map reveals that existence of multiple dominant frequencies through out the tremor period. BMFLC requires an initial period of 1 s for weight stabilization. For surgeon #1 and novice subject #1, it can be correlated from the peaks in the amplitude spectrum shown in Figure 2(a1) about the existence of multiple dominant frequencies throughout the time period in Figure 2(a2). Comparing the time-frequency map with spectrogram shown in Figure 2(a3), the former shows clear distinction in multiple dominant frequencies. In all the subjects involved in our study there existed a bandwidth of 2–5 Hz with several peaks in the amplitude spectrum and several dominant time-frequency components in the time-frequency map.

## 3. Methods

In this section, we first discuss the existing methods on tremor and later propose two improved methods. Existing methods for tremor can be categorized as single frequency based tremor estimation methods and multiple-frequency based tremor estimation methods. Weighted Fourier Linear Combiner (WFLC) [20] and WFLC-Kalman filters [7] rely on single frequency estimation, whereas Bandlimited Multiple-Fourier Linear Combiner (BMFLC) rely on multiple frequency components estimation.

### 3.1. Tremor Estimation

#### 3.1.1. Weighted Fourier Linear Combiner (WFLC)

The WFLC [20,26] algorithm extends the well known Fourier Linear Combiner (FLC) [21] algorithm to also adapt to the time-varying reference signal frequency, using a modification of the LMS algorithm. As FLC only operates at a fixed frequency, the goal of WFLC algorithm is to adapt to periodic signal of unknown frequency, phase and amplitude. The algorithm can be given as

(1) $$x_{\mathrm{rk}}=\{\begin{array}{ll} \text{sin}\;\left(r\sum_{t=0}^{k}\omega_{0_{t}}\right), & 1\leq r\leq M\\ \text{cos}\;\left((r-M)\sum_{t=0}^{k}\omega_{0_{t}}\right), & M+1\;\leq r\leq 2M \end{array}$$

(2) $$\begin{array}{ccc} \varepsilon_{k} & = & s_{k}-{\text{w}}_{k}^{T}{\text{x}}_{k}\\ \omega_{0_{k+1}} & = & \omega_{0_{k}}+2\mu_{0}\varepsilon_{k}\sum_{r=1}^{M}r\left(\omega_{r_{k}}x_{M+r_{k}}-\omega_{M+r_{k}}x_{r_{k}}\right)\\ {\text{w}}_{k+1} & = & {\text{w}}_{k}+2\mu {\text{x}}_{k}\varepsilon_{k} \end{array}$$

where input signal amplitude and phase are estimated by the adaptive vector **w***_k_* similar to FLC, whereas *ω*_0_*k*__ estimates the unknown frequency of the input signal. *μ* and *μ*_0_ are adaptive gain parameters that govern the adaptation process of frequency and amplitude respectively. In usual practice, the combination of WFLC and FLC is employed for tremor filtering. The main advantage of WFLC is that it can adapt to changes in frequency of the signal. However, if the frequency variations are fast enough (signal frequency does not remain constant over time), the performance of WFLC will be degraded.

Recently a two stage algorithm was developed to improve the performance of the WFLC by employing a Kalman Filter (KF) to minimize the estimation error [7]. In the first stage, frequency estimation is performed with WFLC and in the second stage, Kalman Filter estimates the tremor amplitude with the frequency input from WFLC. The accuracy of estimation in this cascade filter completely relies on the estimation accuracy of WFLC. If the tremor signal contains multiple dominant frequencies, the frequency weight does not converge and it effects the overall accuracy of the WFLC-Kalman filter.

Presence of multiple peaks in the fast Fourier transform (FFT) spectrum is the result of modulation of multiple frequency components in tremor. Existing methods FLC and WFLC algorithms adapt to a single frequency present in the incoming signal. For the tremor signal consisting of multiple dominant frequencies closely, the accuracy in tremor estimation decreases with WFLC [6]. In [9], it was demonstrated that for signals with multiple frequencies, the WFLC estimation performance degrades as the gap between the frequencies increases. For this case, the frequency adaption process of the WFLC can never be stabilized and accurate estimation of the tremor signal cannot be attained.

#### 3.1.2. Bandlimited Multiple-Fourier Linear Combiner (BMFLC)

**Figure 3.**
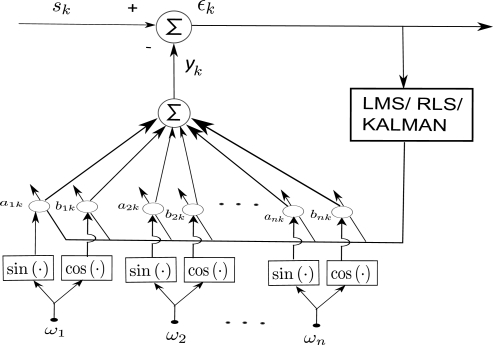
BMFLC Architecture.

To overcome the problems with tremor signals comprising of multiple dominant frequencies, BMFLC [9,22] was recently developed. To estimate the tremor signal in the pre-defined band [*ω*_1_ − *ω_n_*], a series comprising of sine and cosine components are combined to form bandlimited multiple-Fourier Linear Combiner (BMFLC):

(3) $$y_{k}=\sum_{r=1}^{n}a_{\mathrm{rk}}\;\text{sin}(\omega_{r}k)+b_{\mathrm{rk}}\;\text{cos}(\omega_{r}k)$$

where *y_k_* denotes the estimated signal at sampling instant *k*. *a_rk_*, *b_rk_* represents the adaptive weights corresponding to the frequency *ω_r_* at instant *k*. Δ*ω* represents the step size in the frequency band [*ω*_1_*−ω_n_*] and *n* = [*ω*_1_ − *ω_n_*]*/*Δ*ω*. The series only considers “*n*” fundamental frequencies in the band. LMS algorithm [27] is employed to adapt the weights *a_rk_*, *b_rk_* in Equation (3) to the incoming unknown signal. The architecture of the proposed algorithm is shown in Figure 3. The algorithm can be stated as follows:

(4) $${\text{x}}_{k}=\left\{\begin{array}{c} {\left[\begin{array}{cccc} \text{sin}\left(\omega_{1}k\right) & \text{sin}\left(\omega_{2}k\right) & \cdots & \text{sin}\left(\omega_{n}k\right) \end{array}\right]}^{T}\\ {\left[\begin{array}{cccc} \text{cos}\left(\omega_{1}k\right) & \text{cos}\left(\omega_{2}k\right) & \cdots & \text{cos}\left(\omega_{n}k\right) \end{array}\right]}^{T} \end{array}\right\}$$

(5) $${\text{w}}_{k}=\left\{\begin{array}{c} {\left[\begin{array}{cccc} a_{1k} & a_{2k} & \cdots & a_{\mathrm{nk}} \end{array}\right]}^{T}\\ {\left[\begin{array}{cccc} b_{1k} & b_{2k} & \cdots & b_{\mathrm{nk}} \end{array}\right]}^{T} \end{array}\right\}$$

(6) $$y_{k}={\text{w}}_{k}^{T}{\text{x}}_{k}$$

(7) $${ɛ}_{k}=s_{k}-y_{k}$$

LMS update:

(8) $${\text{w}}_{k+1}={\text{w}}_{k}+2\mu {\text{x}}_{k}{ɛ}_{k}$$

where **x***_k_* is the reference input vector, *s_k_* is the reference signal, *ε_k_* represents the error term and *μ* and is an adaptive gain parameter. As shown in architecture, n-FLC’s are combined to form the BMFLC to estimate bandlimited signals. The time constant for convergence can be shown to be $\frac{1}{2\mu }$ [21]. The adaptive gain parameter *μ* can be chosen to have fast convergence without loosing stability.

Input signal amplitude and phase are estimated by the adaptive vector **w***_k_*. Frequency spacing of 0.1–0.5 for Δ*ω* and band of 7–14 Hz is optimum for estimation of tremor. Equations (4–7) represent the BMFLC algorithm, where as Equation (8) is the weight update equation according to LMS algorithm. With the LMS optimization algorithm, the corresponding weights of the individual frequencies adapt to their respective frequency components present in the band of interest. As the frequency components are fixed in the algorithm, any modulated signal can be estimated in the given band with the amplitude weights. Comparing BMFLC with WFLC, the former requires parallel adaptation for frequency and amplitude and hence the amplitude estimate cannot be accurate unless the frequency weight stabilizes.

#### 3.1.3. BMFLC with Recursive Least Squares (BMFLC-RLS)

As LMS algorithm [27] relies on gradient based method for error minimization, the accuracy of the algorithm can be affected by the dynamic changes in the characteristics of the signal. LMS algorithm has only a single adjustable parameter for controlling the convergence rate, namely, the step-size parameter *μ*. To further improve the performance, in this section we combine BMFLC with RLS algorithm.

The recursive least squares algorithm (RLS) [28,29] is an adaptive algorithm that uses successive corrections for the filter coefficients and minimizes weighted least squares error function related to the input signal. RLS algorithm requires the computation of matrix inverse for implementation. To avoid the direct inverse computation, Kalman gain is employed [28]. The inverse is obtained recursively with the Kalman gain vector and updating the inverse of correlation matrix. The update equations for RLS equations are given by

- Compute Kalman gain **K***_k_*
  (9) $${\text{K}}_{k}=\frac{{\text{P}}_{k}{\text{x}}_{k}}{\lambda +{\text{x}}_{k}^{T}{\text{P}}_{k}{\text{x}}_{k}}$$
- Update the BMFLC weights
  (10) $${\text{w}}_{k+1}={\text{w}}_{k}+{\text{K}}_{k}{ɛ}_{k}$$
- Update correlation matrix **P***_k_*
  (11) $${\text{P}}_{k+1}=\frac{1}{\lambda }\left[{\text{P}}_{k}-{\text{K}}_{k}{\text{x}}_{k}^{T}{\text{P}}_{k}\right]$$

The main purpose of the forgetting factor *λ* (typically 0.9 *≤* *λ* *≤* 1) in Equation (9) is to weight the most recent data points more heavily and to allow the prediction coefficients to adapt to time varying statistical characteristics of the data. By replacing the LMS update Equation (8) with above RLS update Equations (9–11), BMFLC based RLS algorithm is formulated.

#### 3.1.4. BMFLC with Kalman Filter (BMFLC-Kalman)

Kalman filter [30–32] is an important tool for estimation of states in dynamic systems with recursive procedure. The formulation of the Kalman filter is generally described in the state-space form. To model the BMFLC in the state-space form, the adaptive weight vector **w***_k_* is considered to be state vector. State transition (weights transition) with no priori information can be modelled as a random walk model:

(12) $${\text{w}}_{k+1}={\text{w}}_{k}+\eta_{k}$$

where *η_k_* is the state error in the state transition. From Equations (6,7), the BMFLC estimation can be re-written in the state-space form as

(13) $$s_{k}={\text{x}}_{k}^{T}{\text{w}}_{k}+v_{k}$$

The above dynamics is in the form linear observation model with **x***_k_*, the reference vector and *v_k_*, the observation error. Equations (12) and (13) form a reduced state-space model. Optimal estimation can be developed for the time varying adaptive weights **w***_k_* in state-space form. We assume that the measurement noise *v_k_* and state noise *η_k_* are uncorrelated, zero mean, Gaussian white noise processes with covariances *R* and **Q**. Even if the assumption of the noise does not hold, it was shown that the Kalman filter [33] can give the minimum mean-squared error within the class of linear estimators. The Kalman filter can be designed to estimate the state of the dynamical system at any time instant *k* with the measurement sequence **s**_1:*k*−1_ = [*s*_1_, *s*_2_, ⋯, *s*_*k*−1_]. In this section, we employ the following notation:

$${\hat{w}}_{k}=\text{E}\left\{{\text{w}}_{k}|s_{1:k-1}\right\}$$

where **E**{·} denotes the expectation. Given the measurement sequence **s**_1:*k*−1_, the estimated state**ŵ***_k_* and the estimated state error covariance **P***_k_* can be obtained by Kalman filter recursively [34]:

- Compute Kalman gain **K***_k_*
  (14) $${\text{K}}_{k}={\text{P}}_{k}{\text{x}}_{k}^{T}{\left({\text{x}}_{k}^{T}{\text{P}}_{k}{\text{x}}_{k}+R\right)}^{-1}$$
- Update BMFLC weights
  (15) $${\hat{\text{w}}}_{k+1}={\hat{\text{w}}}_{k}+{\text{K}}_{k}\left(s_{k}-{\text{x}}_{k}^{T}{\hat{\text{w}}}_{k}\right)$$
- Update covariance matrix
  (16) $${\text{P}}_{k+1}=\left[\text{I}-{\text{K}}_{k}{\text{x}}_{k}\right]{\text{P}}_{k}+\text{Q}$$

with initial conditions **w**_0_ and **P**_0_. **K***_k_* is the Kalman gain updated at each time instant. By replacing the LMS update Equation (8) with above Kalman Filter update Equations (14–16), BMFLC based Kalman Filter is formulated. The BMFLC-RLS and BMFLC-Kalman algorithm does not require the matrix inverse as the BMFLC is modeled in the form of single-output model. The proposed algorithms are computationally fast and are well suited for real-time implementations.

### 3.2. Filtering of Voluntary Movement

**Figure 4.**
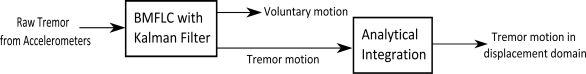
Block diagram for BMFLC-Kalman tremor filtering.

Accurate separation of voluntary motion from raw data is extremely important for successful compensation in robotics applications. To deal with this problem, in our proposed algorithm a bias weight [27] with adaptive gain is introduced to separate the intended motion and drift (low frequency component) from the tremor signal. This avoids the need of pre-filtering (lowpass filter) as required in WFLC and WFLC-Kalman Filters to remove the voluntary motion. By construction, BMFLC can identify the voluntary motion without any pre-filtering and can also obtain the drift-free position from acceleration in a single stage as shown in Figure 4. The algorithm can be modified by adding an extra term *a*_0_ > 0 to track the intentional component in the LMS algorithm as follows:

(17) $${\tilde{\text{x}}}_{k}=\left[\begin{array}{c} {\text{x}}_{k}\\ 1 \end{array}\right]$$

where **x̃***_k_* and **w̃***_k_* = [*a*_1*k*_ ⋯ *a*_*nk*_ *b*_1*k*_ ⋯ *b*_*nk*_ *a*_0*k*_]^*T*^ are the new reference vector and adaptive weight vectors respectively. Since the high frequency components track their respective frequencies, the weight vector corresponding to 1 will adapt to the voluntary motion/drift in the motion. Therefore, the components can obtained as

(18) $$I\left(k\right)=a_{0k}$$

(19) $$T\left(k\right)=\hat{S}\left(k\right)-I\left(k\right)$$

where *Ŝ*(*k*) is the estimated signal with Equation (17). *I*(*k*) and *T*(*k*) represents the intentional and the tremor portions of the signal at the *k^th^* instant respectively.

### 3.3. Calculation of Displacement with Accelerometers

Robotics based surgical devices such as Micron [12,26], rely on accelerometers to sense the tremor and to cancel in real-time during microsurgery. The cancellation of tremor is performed through piezoelectric actuators in displacement domain. As accelerometers only provide the acceleration measurement, numerical integration is required to obtain the position information. Due to the presence of noise and dc bias, the integration drift grows quadratically over time after double integration. To overcome the drift, BMFLC can be employed in the acceleration domain to directly obtain the displacement of the tremor signal [35]. In Equation (3), as the frequency components remain constant, double integration of Equation (3) yields:

(20) $$y_{\mathrm{disp}}=-\sum_{r=1}^{n}\frac{1}{{\left(\omega_{r}\right)}^{2}}\left[a_{\mathrm{rk}}\;\text{sin}\left(\omega_{r}k\right)+b_{\mathrm{rk}}\;\text{cos}\left(\omega_{r}k\right)\right]$$

As the algorithm provides the weight vectors of all the sine and cosine components, the non-drifting position information can be obtained with Equation (20) without the need of numerical integration.

## 4. Results

In this section, we first discuss the separation of voluntary motion with BMFLC-Kalman filter on the raw data recorded during our trails and later compare the performance of all algorithms on the filtered data.

**Figure 5.**
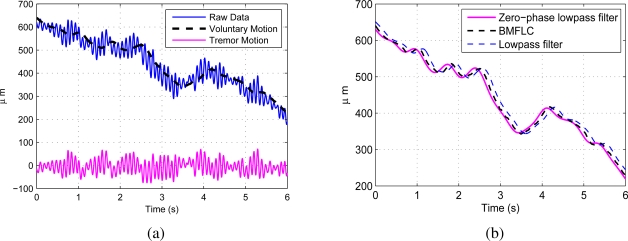
**(a)** Raw data recorded from Subject #4 with tracing task and the identified voluntary motion; **(b)** Comparison with zero-phase lowpass filter and Lowpass filter.

With addition of the extra weight as discussed in Section 3.2, the algorithm tracks the low-frequency component (voluntary movement). The raw data recorded from a healthy subject performing a tracing task is shown in Figure 5(a). The voluntary motion is separated from the raw data with the method discussed in Section 3.5 without any pre-filtering. The identified voluntary motion together with the estimated tremulous motion are shown in the same figure. The proposed method is compared with zero-phase low-pass filtered data and low-pass filtered data with cutoff frequencies 2 Hz and is shown in Figure 5(b). Compared to zero-phase low-pass filter, the proposed method has a delay of 0.082 s with RMSE 4.61 *μm*, whereas the low-pass filter has a delay of 0.2 s with RMSE 17.8 *μm*. It is clearly evident that the voluntary motion identified with BMFLC has less delay and low RMSE compared to low-pass filter. Hence, the proposed method will be more suitable for real-time implementation.

**Figure 6.**
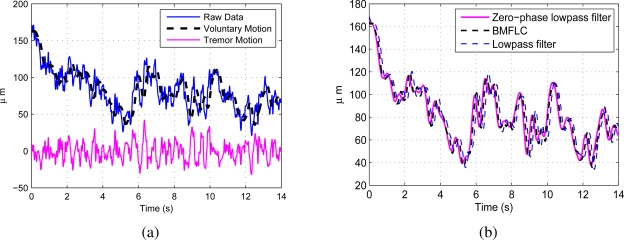
**(a)** Raw data recorded from Surgeon #1 with pointing task and the identified voluntary motion; **(b)** Comparison with Lowpass filter.

Similarly, for the raw data recorded with surgeon #1 performing a pointing task is shown in Figure 6(a) together with the identified voluntary motion. Figure 6(b) shows the comparison with zero-phase low-pass filter and low-pass filter. Compared to zero-phase low-pass filter, proposed method has delay 0.072 s with RMSE 6.63 *μm*, whereas low-pass filter has delay 0.186 s with RMSE 26.5 *μm*.

To evaluate the performance of all algorithms, the tremor data of all subjects is bandpass filtered with zero-phase 5th order butterworth filter having pass band 6–14 Hz. The time-frequency map and FFT in Figure 2(a,b) for surgeon #1 evidently shows the existence of multiple dominant frequencies in the band of 7–11 Hz. The bandpass filtered data is similar to the tremor data identified with the method proposed in Section 3.5. Bandpass filtered signal is essential for estimation with WFLC and WFLC-Kalman Filters [26]. Implementation of this bandpass filter in real-time causes delay and it decreases the accuracy of tremor compensation in real-time applications. For sake of ideal comparison, bandpass filter is employed.

**Figure 7.**
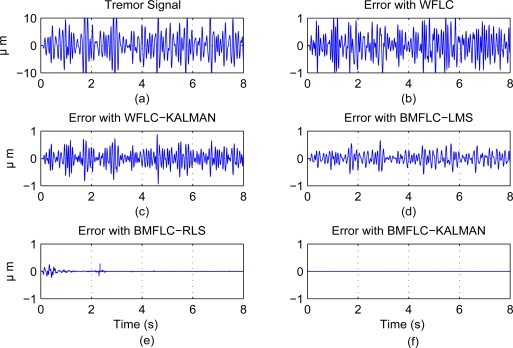
Performance of all algorithms with Surgeon #1 (pointing task).

The following parameters and initial conditions are set for all the algorithms:

- WFLC algorithm: *μ*_0_ = 1.10^−5^, *μ* = 5.10^−4^, *f*_0_ = 7 Hz
- WFLC-Kalman: *μ*_0_ = 1.10^−5^, *μ* = 5.10^−4^, *f*_0_ = 7 Hz, *R* = 0.01, **Q** = 0.01*×***I** and **P**_0_ = 0.01*×***I**
- BMFLC: *ω*_1_ = 2*π* *×* 7, *ω_n_* = 2*π* *×* 14, Δ*ω* = 0.1 and *μ* = 0.01
- BMFLC-RLS: *ω*_1_ = 2*π* *×* 7, *ω_n_* = 2*π* *×* 14, Δ*ω* = 0.1, *λ* = 0.95 and **P**_0_ = 0.1 *×* **I**
- BMFLC-Kalman: *ω*_1_ = 2*π* *×* 7, *ω_n_* = 2*π* *×* 14, Δ*ω* = 0.1, *R* = 0.01, **Q** = 0.01 *×* **I** and **P**_0_ = 0.01 *×* **I**

where **I** is the identity matrix of appropriate dimension. The tremor motion obtained with band-pass filtering is considered for analysis of algorithms and is shown in Figure 7(a). The comparative performance of all the five algorithms for the purpose of illustration are shown in Figure 7. It is clearly evident that BMFLC-RLS and BMFLC-Kalman perform better than the rest of the algorithms. Due to presence of multiple dominant frequencies, frequency weights does not settle and 90% accuracy was obtained. Employing WFLC-Kalman improved the accuracy 93%. BMFLC based algorithms provided 94%, 99% and 99.5% with LMS, RLS, and Kalman filter respectively. It is clearly evident that replacing the LMS update with Kalman filter improves the accuracy in estimation.

To further quantify the performance of all algorithms, the data recorded for two tasks (pointing task and tracing task with two trails/task) is considered for analysis. The analysis is performed separately for surgeons and novice subjects. All the algorithms are prediction based and only rely on output measurement *s_k_* at instant *k* to predict the *y_k_*_+1_ estimate.

Table 1 summarizes the performance of all the five algorithms for 6 novice subjects and 6 microsurgeons for all trails. The average RMSE and standard deviation are provided in the table. Among the five algorithms, BMFLC-Kalman provides the best estimate for tremor with the least RMSE.

## 5. Discussion

Accuracy of cancellation of tremor in robotics instruments mainly depends on separation of tremulous motion from the raw data. It is necessary to develop novel methods for accurate filtering and estimation of physiological tremor in real-time for surgical applications. The proposed algorithm with a constant weight filters the low-frequency component, *i.e.*, voluntary motion from the raw data and separates the tremor motion. The performance is similar to that of a lowpass filter much less delay as observed in Figures 6(b) and 5(b). The proposed algorithm can be applied in a single-stage to identify the voluntary motion and the tremor motion.

As healthy subjects tremor characteristics display a band with multiple dominant frequencies, WFLC based algorithms fail to model tremor accurately. The single frequency component in WFLC has to adapt to all the frequency changes in the signal and high accuracy cannot be obtained. An improvement in the performance can be seen by integrating WFLC with Kalman filter. BMFLC based algorithms outperform the rest of the algorithms due to inherent nature of tremor with multiple dominant frequencies. It should be noted that WFLC based methods can be employed for pathological tremor filtering as the tremor consists of a single dominant frequency.

**Figure 8.**
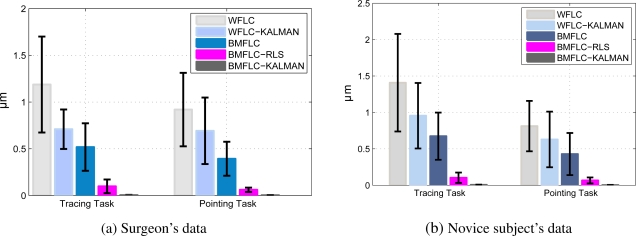
Average RMS tracking error (*μm*) for all algorithms with tracing and pointing tasks; error bars represent standard deviation around mean.

As tracing task involves more control, subject tend to display larger variations in tremor amplitude compared to pointing task. To study the difference in performance for pointing and tracing tasks, the analysis is performed separately for two tasks. The error bars with mean and standard deviation are shown in Figure 8(a,b). As pointing task is less complex than the tracing task, the average RMS estimation errors for tracing tasks are higher than that of pointing tasks.

As part of our continuing research in developing smart surgical device such as Micron [12,26], with accelerometers, the algorithms are developed for cancellation of tremor in real-time with accelerometers. 3-DOF accelerometers based sensing is employed to provide tip position of X, Y and Z axis separately. The acceleration data from accelerometers can be analytically converted to displacement with the proposed algorithm. The proposed method will be tested for 3-DOF cancellation of tremor by cancellation of tremor in all three axes separately. Microsurgery involves a lot of complex gestures, e.g., a intentional sudden jerk caused by a surgeon is a huge challenge for identification and filtering in real-time. Further research is required to deal with complex gestures and sudden jerks involved in microsurgery.

## 6. Conclusions

This paper presents an improved single stage algorithm for estimating tremor for data sensed with accelerometers. The voluntary motion and involuntary motion can be separated from raw data accurately with the proposed method. Existing method BMFLC with LMS algorithm is improved by replacing LMS algorithm with Kalman filter. To analyze the performance of all the algorithms, a comprehensive comparative study is conducted on the data recorded from 6 healthy subjects and 6 microsurgeons. To highlight the performance of the proposed methods, we evaluate both the state of the art algorithms with the two novel-methods developed in this paper. The proposed methods BMFLC-Kalman and BMFLC RLS performed better than the existing methods WFLC, WFLC-Kalman and BMFLC-LMS. Among the five algorithms BMFLC-Kalman performed better producing an accurate estimate of tremor with an average RMS error of 0.003 *±* 0.002 (*μ*m) and is more suited for real-time estimation. With the proposed algorithm, accelerometers data can be used to obtain the position information.

## Figures and Tables

**Table 1.** Average RMS error on all trails for tremor estimation algorithms.

**Subjects**	**Average RMS error** (*μ m*) **and standard deviation**				
	WFLC	WFLC-Kalman	BMFLC	BMFLC-RLS	BMFLC-Kalman
6 Novice subjects	1.065 *±* 0.578	0.747 *±* 0.43	0.512 *±* 0.303	0.08 *±* 0.05	0.004 *±* 0.002
6 Surgeons	0.956 *±* 0.441	0.632 *±* 0.263	0.408 *±* 0.201	0.076 *±* 0.051	0.003 *±* 0.002
